# COVID-19 pandemic and the international classification of functioning in multiple system atrophy: a cross-sectional, nationwide survey in Japan

**DOI:** 10.1038/s41598-022-18533-w

**Published:** 2022-08-19

**Authors:** Koshiro Haruyama, Michiyuki Kawakami, Ichiro Miyai, Shuko Nojiri, Toshiyuki Fujiwara

**Affiliations:** 1 Department of Physical Therapy, Faculty of Health Science, Juntendo University, Tokyo, Japan; 2 Department of Rehabilitation Medicine, Juntendo University Graduate School of Medicine, Tokyo, Japan; 3 Department of Rehabilitation Medicine, School of Medicine, Keio University, Tokyo, Japan; 4 Neurorehabilitation Research Institute, Morinomiya Hospital, Osaka, Japan; 5 Medical Technology Innovation Center, Juntendo University, Tokyo, Japan

**Keywords:** Diseases, Health care, Medical research, Neurology

## Abstract

The present study aimed to determine the magnitude of and risk factors for the effects of the COVID-19 pandemic on the international classification of functioning, disability and health (ICF) in patients with multiple system atrophy (PwMSA). The study was part of a cross-sectional, nationwide, multipurpose mail survey for Japanese PwMSA from October to December, 2020. The primary outcome was the impact of the early COVID-19 pandemic on ICF functioning, consisting of body function, activity, and participation. Age, sex, disease type, disease duration, and dwelling place were asked as participants’ characteristics, and the multiple system impairment questionnaire (MSIQ), patient health questionnaire-2, modified rankin scale, barthel index, life-space assessment (LSA), and EuroQoL were examined. Multivariate logistic regression analyses were performed to identify independent risk factors for a worse function score due to the COVID-19 pandemic for each ICF functioning domain. A total of 155 patients (mean age 65.6 [SD 8.1] years; 43.9% women; mean disease duration 8.0 [SD 6.2] years; 65% MSA with cerebellar ataxia, 13% MSA with parkinsonism, 9% MSA with predominant autonomic features) were analyzed. Of the ICF functioning domains, the respondents reported that the early COVID-19 pandemic affected body function in 17.4%, activity in 17.6%, and participation in 46.0%. The adjusted multivariate model identified MSIQ and LSA as the two variables that independently contributed to all domains. The COVID-19 pandemic affected ICF functioning of PwMSA in Japan, and the severity of disease-related impairments and a large daily living space were common risk factors. These results help support the focus on patient characteristics for medical and social welfare support.

## Introduction

Coronavirus disease 2019 (COVID-19) was declared a pandemic by the World Health Organization on March 11, 2020^1^. The Government of Japan responded to the first wave of infection by declaring “a nationwide state of emergency” on April 16, lasting until May 25, and requested that the public refrain from leaving home unless necessary. Due to legal restrictions in Japan, a mitigation strategy was adopted, and a forced lockdown was not implemented. As a result, Japan experienced the third wave of the COVID-19 pandemic in November 2020, during this survey, following the first wave in April and the second wave in July. The spread of the COVID-19 pandemic had a wide range of consequences, including not only physical limitations, but also psychological and social health effects^2,3^. In a large web-based survey conducted in Japan in November 2020, about 18% of the general adult population reported that their global health had deteriorated, and their lifestyle had changed negatively since the start of the COVID-19 pandemic^4^. Restrictions on social participation due to the COVID-19 pandemic seem to lead to an apparent decrease in physical activity in adult and elderly populations^4,5^. Furthermore, it has been noted that people with pre-existing physical and mental illnesses may experience reduced social contact, leading to poorer health and quality of life (QOL)^6,7^. In particular, patients with neurological disorders need to be more sensitive to infection prevention^8,9^. However, there is still a lack of evidence-based information on the effects of the COVID-19 pandemic on patient populations^10^.

Multiple system atrophy (MSA) is a sporadic orphan disease characterized by progressive autonomic dysfunction, parkinsonian features, and cerebellar and pyramidal features in various combinations^11^. The two main subtypes are MSA with predominant parkinsonism (MSA-P) and MSA with predominant cerebellar ataxia (MSA-C)^12^. The majority of patients with MSA (PwMSA) diagnosed in Europe and North America have MSA-P, whereas those in Japan have MSA-C^13–15^. The subtype characterized by autonomic dysfunction of much greater severity than other symptoms is sometimes referred to as MSA with predominant autonomic features (MSA-A)^16^. The estimated point prevalence is 3.4 to 4.9 cases per 100,000 population, increasing to 7.8 per 100,000 among persons older than 40 years^17^. Approximately 50% of patients require walking aids within 3 years after the onset of motor symptoms, 60% require a wheelchair after 5 years, median time before the patient is bedridden is 6 to 8 years, and mean survival is 6 to 10 years^11^. Previous small-scale reports have shown that the COVID-19 pandemic has affected global health and symptom progression in PwMSA^18,19^. An international panel of ataxia experts has suggested that it is necessary for patients with cerebellar ataxia to take measures to avoid contact with COVID-19 during epidemics^20^.

“Functioning” is recognized as the third health indicator alongside mortality and morbidity, even in the COVID-19 pandemic^21^. Functioning is defined as an umbrella term in the international classification of functioning, disability and health (ICF) for body functions and structures, activities, and participation^22^. The effect of the COVID-19 pandemic on PwMSA has not been studied from the perspective of ICF domains. The theoretical assumption of functioning is that body function, activity, and participation affect each other interactively^22^, but the relevance of the disability structure under the COVID-19 pandemic is unclear. In addition to the nationwide long-term curtailment of going out, many patients are believed to have restricted themselves from going out, especially since elderly persons and persons with underlying diseases were actively warned to do so by the government. Given such a social situation, it can be assumed that the effect of the COVID-19 pandemic will first be seen in the domain of social participation, followed by activity and body function.

The aim of the present study was to determine which domains of the ICF would be affected by the early COVID-19 pandemic in PwMSA, and which patients with which clinical conditions would be more susceptible to decreased function. Our simple hypothesis was that participation is most directly affected by the COVID-19 pandemic, followed by activity and body function. A further hypothesis was that participants with some form of peer or social support, despite their severe functional impairment, are the most affected. Thus, the specific purpose of this study was to determine the magnitude of and risk factors for the effects of the COVID-19 pandemic on the ICF domains of body function, activity, and participation in PwMSA.

## Methods

### Study design

This study was part of a cross-sectional, nationwide, multipurpose, mail survey of Japanese PwMSA from October to December, 2020. The data reflect the effects of the early COVID-19 pandemic, corresponding to 8 to 9 months after the global pandemic was declared. Prior to this study, we reported another study of aspects of social services in Japan^23^, and all 155 participants in the present study were also included in that analysis.

### Standard protocol approvals, registrations, and patient consents

The study received approval from the ethics committee of the Faculty of Health Science of Juntendo University (Approval Number 20-012) and was performed in accordance with the Declaration of Helsinki. The study followed the Strengthening the Reporting of Observational Studies in Epidemiology (STROBE) guidelines and the American Association for Public Opinion Research (AAPOR) reporting guideline.

The survey was anonymous, and confidentiality of information was assured. Informed consent was obtained from all participants by providing a written explanation of the study and having them return the questionnaire form. Because it was anticipated that certain patients did not want their responses to be included in the study, their return of a letter of intent to refuse to respond was also accepted.

### Participants

The participants of this study were respondents to a survey of all members of the Japanese spinocerebellar degeneration and MSA patient association (https://scdmsa.tokyo/). This is the largest nonprofit organization patient group in Japan and is composed of volunteers. However, the members may not be the patients themselves, because they may be individuals who agree with the purpose of the association. MSA was self-reported by the patients in this study, but the diagnosis of MSA in Japan is made by specialists according to the common diagnostic criteria specified by the Ministry of Health, Labour and Welfare for registration of designated intractable diseases. The criteria are based on the second consensus statement on the diagnosis of MSA^12^. It was unlikely that an individual belonging to a patient association of rare diseases would self-report without it being based the correct diagnosis. The exclusion criteria for this study were those who were not PwMSA, and those who refused to respond or had incomplete answers to all the questions related to COVID-19.

### Procedure and outcomes

Questionnaires were mailed to all members using the address database used to send regular patient association mailings. The relevant questions consisted of original COVID-19-related items and background information items that did not reflect COVID-19. The primary outcome was the effect of the COVID-19 pandemic on ICF functioning. The domains of ICF functioning consisted of body function, activity, and participation, and these definitions were in accordance with the WHO^22^. The self-perception of ICF functioning during the pandemic was assessed through the patient’s global impression at the time of response. Participants were asked to rate the impact score on each ICF functioning associated with COVID-19 on a 7-point scale: strongly unaffected =  − 3, moderately unaffected =  − 2, slightly unaffected =  − 1, undecided = 0, slightly affected =  + 1, moderately affected =  + 2, and strongly affected =  + 3. Of these, responses “− 1 to − 3” were classified as “Unaffected”, and responses of “+ 1 to + 3” score were classified as “Affected”, with the latter responses defined as COVID-19-related decline of ICF functioning. Thus, if domains of ICF functioning (body function, activity, and participation) were affected, the terms impairment, activity limitation, and participation restriction were used, respectively. The infection status and behavioral effects of COVID-19 were also included in the survey.

Participant were asked about age, sex, disease type, disease duration, and dwelling place (home, long-term care facility, hospitalization), and the multiple system impairment questionnaire (MSIQ), patient health questionnaire-2 (PHQ-2), modified rankin scale (mRS) score, barthel index (BI), life-space assessment (LSA), and EuroQoL (EQ) were examined. Of these, the clinical assessment indices were based on self-reported responses of normal conditions before the COVID-19 pandemic. The MSIQ for comprehensive scoring of the severity of disease-related impairments and the PHQ-2 as a screening tool for depression reflect body function as a baseline. The mRS score was used as a simple indicator of independence level, and the BI was used for comprehensive scoring of basic activities of daily living (ADL) assessment, reflecting activity as a baseline. The LSA was used for comprehensive scoring of the extent of daily living space, and the EQ was used as a global assessment of health-related QOL, and they reflected participation as a baseline.

The MSIQ was developed specifically for this study and was scored on a self-report basis for 22 impairments that may occur in MSA (Online Resource 1). All impairments were described in writing to ensure specific understanding. It consists of a total of 22 items: ataxia, muscle weakness/atrophy, muscle rigidity, spasticity, balance disorder, postural abnormality, decreased endurance, fatigue, pain, numbness, sensory disturbance, tremor, involuntary movements, orthostatic hypotension, poor sleep, respiratory disturbance, speech/dysarthria, dysphagia, visual impairment, urinary impairment, voiding impairment, and cognitive impairment. Each impairment was scored as 0 (no impairment) to 3 (severe impairment). Thus, the maximum score of 66 is the most severe impairment, and a score of 0 is the complete absence of impairments.

The PHQ-2 for depression screening^24^ is a shortened version of the PHQ-9^25^. It consists of two items with a score of 0–3 each, with 0 being normal and 6 being the most severe^24^. The PHQ-2 was used instead of the PHQ-9, which contains a motor-related item^26^.

The mRS was originally developed as an assessment grade for disability or dependence in the ADL of stroke patients. Today, it is widely used in patients with neurological diseases. The score was rated on a six-point scale from 0 (asymptomatic) to 5 (severe disability)^27^.

The BI evaluates the performance of basic ADL, such as feeding, personal hygiene, bathing, and dressing on a scale of 0–100. A higher number reflects a greater ability to function independently and has the advantage of being applicable to self-assessment and direct administration^28^. To allow for self-assessment by mail, the content of previous studies was used^29^.

The LSA is a self-report measure to summarize the distance (five distance levels ranging from room to out of town) and frequency (five frequency levels ranging from not at all to every day) an individual travels in a given period of time. The results are calculated by the LSA score, which is 120 for the most active^30^.

The EQ is a comprehensive measure of health-related QOL that is used worldwide^31^. In this study, the official Japanese version of the EQ five-dimension five-level questionnaire (EQ-5D-5L) was used, and an index value was calculated, with 1 the highest and 0 the lowest^32^.

In summary, to clarify the effect of the COVID-19 pandemic on MSA, ICF functioning during the pandemic was defined as the main outcome, potential predictors of functioning decline were defined as MSIQ, PHQ-2, mRS, BI, LSA, and EQ, and the potential confounders were age, sex, disease duration, and dwelling place.

### Statistical analyses

Descriptive statistics are presented for demographic variables and functional outcomes during the COVID-19 pandemic for PwMSA. Continuous variables are presented as means (standard deviation) and categorical variables as numbers (%). Spearman’s product rate correlation coefficients were calculated for the associations between ICF functioning scores related to the COVID-19 pandemic. To compare the affected patients with the unaffected patients on ICF functioning, group comparisons were performed using Student’s *t-*test for numerical variables, the Mann–Whitney U test for ordinal variables, and the χ^2^ test when appropriate for categorical variables. A score of 0 was excluded from the analysis in that domain. Among the data of selected patients, missing values were excluded only for that item.

Univariate logistic regression analyses were performed on the identified variables to assess the potential risk factors for affected functioning domains during the COVID-19 pandemic. The dependent variable was a dummy variable that was set to 0 for unaffected (− 3 to − 1) and 1 for affected (+ 1 to + 3) for each functioning domain, and the independent variables were the MSIQ, PHQ-2, mRS, BI, LSA, and EQ. Similar analyses were performed for each domain of ICF functioning (impairment, activity limitation, and participation restriction). The associations between risk factors and outcomes are presented as odds ratios (ORs) and 95% confidence intervals (CIs). Finally, multivariate logistic regression analyses were performed to identify independent risk factors for affected function scores during the COVID-19 pandemic for each domain of ICF functioning. Age, sex, disease duration, and dwelling place were forced into the model as adjustment factors, and a stepwise variable increase method (likelihood ratio) was used with independent variables identified on univariate logistic regression analysis. ORs and 95% CIs are presented after adjustment for confounders, including age, sex, disease duration, and dwelling place. For multivariate analysis only, participants with even one missing value were excluded from the analysis, and only complete data were analyzed.

Data analysis was performed using SPSS statistical software version 27.0 (IBM Corp). The significance level was set at α = 0.05, and all tests were 2-tailed.

### Ethical approval

All procedures performed in the study were approved by the ethics committee of the Faculty of Health Science of Juntendo University (approval number 20-012).

### Informed consent

Informed consent was obtained from all participants by providing a written explanation of the study and by their return of the questionnaire form.

## Results

### Demographic characteristics

**Figure 1:**
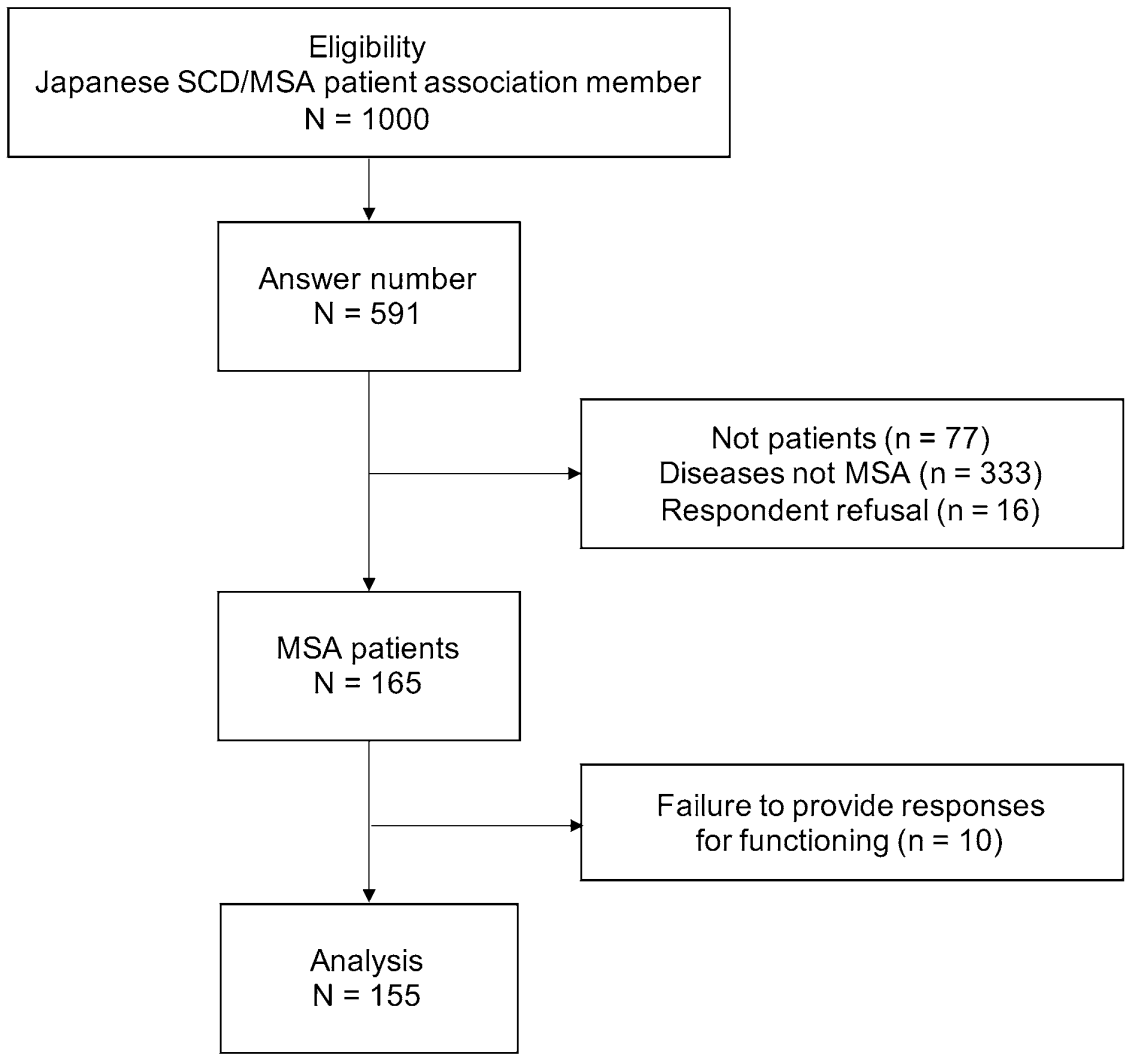
Recruitment flow of patients with MSA.

Questionnaires were mailed to 1000 participants, and 591 were returned. Based on the criteria, 165 patients met the eligibility criteria. In addition, data regarding ICF functioning were missing for 10 patients (6.1%). The outcome rates based on the AAPOR statement were response rate 2 of 58.2%, cooperation rate 4 of 97.4%, Refusal Rate 1 of 1.6%, and Contact Rate 1 of 59.7%. The final analysis included 155 PwMSA (Fig. 1).

Demographic characteristics are presented in Table 1. The mean age was 65.6 (8.1) years, 43.9% were women, and the mean disease duration was 8.0 (6.2) years. The specific diagnoses of MSA were MSA-C in 100, MSA-P in 20, MSA-A in 14, and unknown or difficult to classify in 21. The dwelling place was home in 92.3%, with the rest living in a long-term care facility or hospitalized. There were no reports of COVID-19 infection among the participants themselves or those living with them. Staying at home (self-quarantine) due to the COVID-19 pandemic was practiced by 88.5%, whereas 21.4% restricted their use of rehabilitation services as an indirect effect of the COVID-19 pandemic.

**Table 1:** Patients’ characteristics.

	N	Mean (SD)/no. (%)
Age (years)	155	65.6 (8.1)
Female	155	68 (43.9)
**Diagnosis**	155	
MSA-C		100 (64.5)
MSA-P		20 (12.9)
MSA-A		14 (9.0)
Unknown		21 (13.5)
Disease duration [years]	149	8.0 (6.2)
Status [home/LTCF/hospital]	154	143 (92.9)/9 (5.8)/2 (1.3)
Staying at home [yes/no]	130	115 (88.5)/15 (11.5)
Limitation of rehabilitation services [yes/no]	117	25 (21.4)/92 (78.6)

*MSA* multiple system atrophy, *MSA-C* MSA with predominant cerebellar ataxia, *MSA-P* MSA with predominant parkinsonism, *MSA-A* MSA with predominant autonomic features, *LTCF* long-term care facility.

### ICF domains

**Figure 2:**
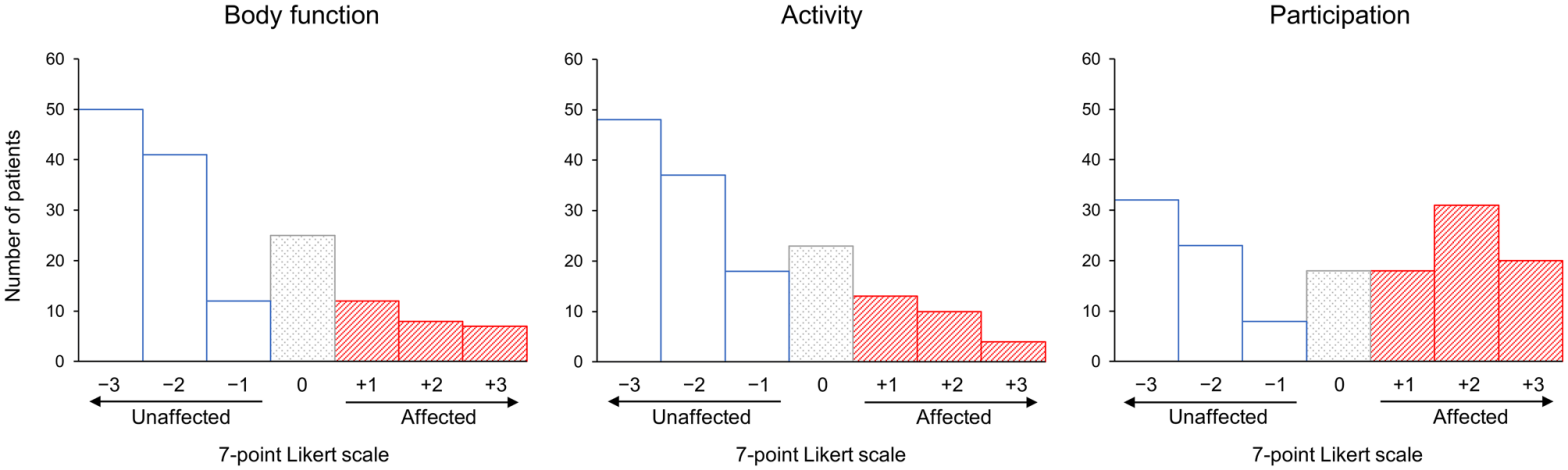
Effect of the COVID-19 pandemic on ICF functioning domains. A color has been assigned to each of the three groups: + 1 to + 3 for COVID-19 pandemic-affected (red oblique bar), − 1 to − 3 for unaffected (white solid bar), and 0 for neither (gray dotted bar).

Regarding the effect of the COVID-19 pandemic, 46.0% reported participation restriction. In contrast, impairment and activity limitation were reported by 17.4% and 17.6%, respectively (Fig. 2). Body function and activity had the largest percentages of participants who reported being strongly unaffected.

### Correlations among ICF functioning

A high correlation was found between the body function and activity domains (r = 0.85). There were moderate correlations with the participation domain of the domains of body function (r = 0.44) and activity (r = 0.45).

### The affected/unaffected group comparison

The comparison between the affected and unaffected groups in each functioning domain showed some specific characteristics (Table 2). There were no significant differences in age, sex, or duration of disease across all comparisons. The MSIQ score was significantly higher in the affected group in the body function and activity domains (p < 0.01 and p = 0.02). The LSA score was significantly higher in the affected group in the activity and participation domains (p = 0.04 and p < 0.01).

**Table 2:** Comparison of patients affected and unaffected by the COVID-19 pandemic.

	Body function			Activity			Participation		
	Affected (n = 27)	Unaffected (n = 103)	p-value	Affected (n = 27)	Unaffected (n = 103)	p-value	Affected (n = 69)	Unaffected (n = 103)	p-value
Age (y)	65.0 (8.8)	66.0 (8.1)	0.60	64.9 (9.0)	65.8 (7.6)	0.60	64.6 (7.9)	67.3 (7.9)	0.05
Sex [female/male]	9/18	48/55	0.22	11/16	47/56	0.65	29/40	32/31	0.31
Disease duration (y)	5.2 (3.6, 9.2)	6.3 (4.0, 10.0)	0.53	5.6 (3.4, 11.4)	6.5 (4.1, 10.0)	0.58	6.6 (3.5, 10.8)	6.4 (4.0, 9.8)	0.91
MSIQ score [0–66]	40.1 (14.0)	32.1 (13.5)	< 0.01	39.3 (15.4)	32.0 (13.0)	0.02	34.9 (15.2)	31.8 (12.0)	0.26
PHQ-2 score [0–6]	2 (0, 3)	2 (0, 4)	0.84	2 (1, 4)	2 (0, 3)	0.27	2 (0, 4)	2 (0, 3)	0.11
mRS score [0–5]	4 (4, 5)	4 (4, 5)	0.93	4 (4, 5)	4 (4, 5)	0.67	4 (3, 5)	4 (4, 5)	0.15
BI [0–100]	43 (0, 95)	38 (0, 90)	0.94	58 (0, 95)	43 (0, 91)	0.83	43 (0, 95)	38 (0, 90)	0.64
LSA score [0–120]	27 (17, 36)	20 (8, 32)	0.09	25 (17, 32)	20 (9, 34)	0.16	30 (16, 41)	18 (4, 29)	0.01
EQ value [0.00–1.00]	0.36 (0.22)	0.38 (0.23)	0.68	0.38 (0.21)	0.38 (0.23)	0.90	0.40 (0.25)	0.35 (0.21)	0.28

Data are presented as means (standard deviations) for numerical variables that showed a normal distribution, median (25th, 75th percentiles) for ordinal variables and numerical variables that did not show a normal distribution, and actual numbers for categorical variables.

*MSIQ* multiple system impairment questionnaire, *PHQ-2* patient health questionnaire-2, *mRS* modified rankin scale, *BI* barthel index, *LSA* life-space assessment, *EQ* EuroQoL.

### Logistic regression analyses

The set of complete data without missing values (110 PwMSA) was used in the regression models, and participants with functioning scores of 0 were excluded from the analysis. The univariate logistic regression model showed that MSIQ was significantly related to impairment and activity limitation, and the LSA score was significantly related to activity limitation and participation restriction. The multivariate model identified the MSIQ score and the LSA score as the two variables that independently contributed to all domains. In terms of ORs for the MSIQ score and the LSA score, they were 1.083 (95% CI 1.032–1.136) and 1.030 (95% CI 1.006–1.054) for impairment, 1.073 (95% CI 1.025–1.123) and 1.035 (95% CI 1.011–1.059) for activity limitation, and 1.052 (95% CI 1.014–1.091) and 1.048 (95% CI 1.021–1.076) for participation restriction, respectively (Table 3). Throughout all analyses, basic characteristics such as age and sex were not identified as sole risk factors.

**Table 3:** Univariate and multivariate logistic regression analyses.

Dependent variable	Independent variable	Univariate analysis		Multivariate analysis^a^	
		OR (95% CI)	p-value	OR (95% CI)	p-value
Impairment	MSIQ score	1.045 (1.011–1.080)	0.010*	1.083 (1.032–1.136)	0.001*
	PHQ-2 score	1.019 (0.826–1.256)	0.862		
	mRS score	1.043 (0.688–1.581)	0.843		
	BI	1.000 (0.990–1.011)	0.962		
	LSA score	1.013 (0.998–1.028)	0.092	1.030 (1.006–1.054)	0.015*
	EQ value	0.688 (0.101–4.708)	0.703		
Activity limitation	MSIQ score	1.041 (1.007–1.075)	0.017*	1.073 (1.025–1.123)	0.003*
	PHQ-2 score	1.108 (0.900–1.364)	0.335		
	mRS score	1.077 (0.700–1.657)	0.735		
	BI	1.001 (0.991–1.012)	0.787		
	LSA score	1.016 (1.000–1.031)	0.046*	1.035 (1.011–1.059)	0.004*
	EQ value	0.912 (0.126–6.582)	0.927		
Participation restriction	MSIQ score	1.015 (0.989–1.041)	0.256	1.052 (1.014–1.091)	0.008*
	PHQ-2 score	1.153 (0.961–1.383)	0.124		
	mRS score	0.743 (0.525–1.050)	0.093		
	BI	1.003 (0.994–1.011)	0.552		
	LSA score	1.030 (1.011–1.049)	0.002*	1.048 (1.021–1.076)	< 0.001*
	EQ value	2.371 (0.491–11.443)	0.283		

*CI* confidence interval, *MSIQ* multiple system impairment questionnaire, *PHQ-2* patient health questionnaire-2, *mRS* modified rankin scale, *BI* barthel index, *LSA* life-space assessment, *EQ* EuroQoL.

^a^Adjusted for age, sex, disease duration, and dwelling place.

## Discussion

This study identified the effect of the early COVID-19 pandemic on ICF functioning and related factors in PwMSA based on Japanese nationwide data. It should be emphasized that the present survey was not an assessment of the full COVID-19 pandemic, but rather of only the early stages of the pandemic, when strict infection control strategies were implemented in each country. As expected, participation restriction was reported by about half of the patients. This result is consistent with the hypothesis that participation is the most directly affected by the COVID-19 pandemic, with limited effects on body function and activity. The focal question was to determine in which patients not only was there participation restriction, but also other decreases in functioning. In particular, severity of disease-related impairments (measured by the MSIQ) and the extent of daily living space (measured by the LSA) affected ICF functioning during the COVID-19 pandemic. A high MSIQ score and a high LSA score were independent risk factors for decreased ICF functioning in all aspects. Compared to previous reports^13,33^, the characteristics of the patients in the present study were higher severity of disease in the participants when referring to the mRS and the BI, as well as higher mean age and longer duration of disease. However, regression analysis showed that these factors and health-related QOL were not associated with COVID-19. These findings can be embodied in the patient characteristics indirectly affected by COVID-19. Unlike the gap between the participation and other domains in the COVID-19 pandemic, patients with a similar clinical status were consistently affected in their overall functioning.

The correlation between participation and the other domains was moderate, indicating that participation restriction caused by the COVID-19 pandemic was not necessarily linked to impairment or activity limitation. On the other hand, the correlation between impairment and activity limitation was very high, and they were similar domains that were almost equally affected. The basic concept of ICF domains is that they are interrelated, and in fact, mild to moderate correlation coefficients between domains have been reported in several populations^34–36^. One possible cause for the characteristic gap among ICFs could be the benefit of the lack of strict lockdowns in Japan. About 90% of respondents reported refraining from going out, whereas only 27% of patients who received rehabilitation reported restrictions of rehabilitation. For example, in Italy during the lockdown, rehabilitation was interrupted in 72% of Friedreich’s ataxia patients^37^ and up to 82% of MS patients^38^. Suspension of hospital treatments or physical therapy, reported in 41% of Parkinson’s disease patients and 43% of hereditary spastic paraplegia and spinocerebellar ataxia patients, was also correlated with subjective worsening of neurological symptoms^7^. In addition, worsening of self-reported health status due to usual rehabilitation limitations was reported in 68.8–82.4% of multiple sclerosis patients with mild to severe disability^39^ and in more than 50% of PwMSA^18^. In contrast to these reports, the fact that necessary rehabilitation services were continued in about 80% of the patients in Japan may have acted to maintain body function^23^.

Consistently identified by regression analysis, the MSIQ was a measure of impairment scored by summing the number and severity of disease-related symptoms. Although whether impairment level even affects the participation domain is controversial, several previous studies have suggested that impairment has a direct association with health-related QOL^40,41^. The relationship between PwMSA disease-related impairments and the COVID-19 pandemic has not been fully elucidated^18^, but there are reports that the disability stage of multiple sclerosis and the progression of disease stage of cerebellar ataxias affect health status^39,42^. Because MSA can cause a wide range of symptoms, including autonomic dysfunction, these findings emphasize the importance of confirming the severity of comprehensive impairments rather than a single activity grade. Furthermore, it was found that the extent of the life space was a risk factor along with a high MSIQ. It has been reported that PwMSA require more care than patients with other neurological diseases^43^, and given the high mRS scores of the participants in the present study (median 4), it is likely that many of them required home care. This result was similar to the hypothesis that participants who maintained their life space with some peer or social support despite severe impairment are the most affected. It is likely that patients who used home care for activity and participation were directly affected by the limitations on caregiving capacity and social services due to the COVID-19 pandemic^44^. In addition, it is clear that high LSA was affected by the “stay at home” recommendation for the pandemic. Limitations in activities outside the home and social interactions spill over into disuse functional impairment and activity limitation^4–7,45^. When managing PwMSA at any level of functioning, attention should be paid to the extent of their living space, as well as disease severity.

### Study strengths and limitations

The main strength was that a large, cross-sectional study confirmed the effect of the pandemic on PwMSA and further identified patient attributes associated with a risk of decreased functioning. This will help to identify potential patients that should be targeted by social services and public policy during or after the COVID-19 pandemic. Specifically, it provides a rationale for monitoring symptom severity and daily living space and for implementing ongoing clinical and community care.

There are several limitations to this study. First, this survey was limited to a single organization during the early COVID-19 pandemic, which led to selection bias. Specifically, most of the cases lived in their homes, so that more advanced cases, such as those living in long-term care facilities or requiring hospitalization, or individuals with less social support not belonging to the association were not well represented in this analysis. Second, the MSIQ used was newly created, and its validity and reliability have not been confirmed. Because of these restrictions, it was used only as a tool to comprehensively measure symptom severity in the present study. Similarly, changes in ICF functioning after the outbreak of COVID-19 were assessed with a questionnaire and not a clinical examination by neurologists, which may result in both underestimation and overestimation. Third, the limited results of PwMSA were not compared to those of healthy or other patient populations. An earlier report using the same patient association database showed a relatively large reduction of ICF functioning in patients with spinocerebellar degeneration, which was a more actionable population assessed by mRS^23^. The impact of COVID-19 may be greater in patient populations with mild activity levels that are more susceptible to participation constraints. Finally, it is not appropriate to generalize the Japanese data to countries or cities with a strict lockdown. Therefore, the present results need to be interpreted by considering the relationship between behavioral status and social systems during the COVID-19 pandemic in different countries and regions. The strategies of countries against the pandemic have been heterogeneous, and not all countries necessarily had full or partial lockdowns^46,47^. Japan’s mitigation strategy, which reflects the current global situation, suggests that the impact of the COVID-19 pandemic on certain patient populations may be limited in the absence of a strict lockdown strategy. Despite these limitations, we are convinced that this patient-oriented survey is an important contribution to understanding the patient situation during the mitigation strategy for the long COVID-19 pandemic.

## Conclusion

The COVID-19 pandemic affected ICF functioning of PwMSA in Japan, particularly in the participation domain, with approximately 45% of PwMSA affected. The severity of disease-related impairments measured by the MSIQ was a common risk factor for decreased ICF functioning in MSA. In addition to the MSIQ, a large extent of daily living space measured by the LSA was a risk factor for a COVID-19 pandemic-induced decrease in ICF functioning. These results help to build a rationale for the social effect of the COVID-19 pandemic on patients with neurodegenerative diseases and to support the focus on patients’ characteristics for medical and social welfare support.

## Supplementary Information

Supplementary Information.

## Data Availability

The datasets generated and analyzed during the current study are available from the corresponding author on reasonable request.

## References

[1] The World Health Organization. *Coronavirus Disease (COVID-19) Pandemic*. https://www.who.int/emergencies/diseases/novel-coronavirus-2019 (2020) (Accessed 30 August 2021).

[2] Li S, Wang Y, Xue J, Zhao N, Zhu T (2020). The impact of COVID-19 epidemic declaration on psychological consequences: A study on active Weibo users. Int. J. Environ. Res. Public Health.

[3] Cai G, Lin Y, Lu Y, He F, Morita K, Yamamoto T, Aoyagi K, Taguri T, Hu Z, Alias H, Danaee M, Wong LP (2021). Behavioural responses and anxiety symptoms during the coronavirus disease 2019 (COVID-19) pandemic in Japan: A large scale cross-sectional study. J. Psychiatr. Res..

[4] Suka M, Yamauchi T, Yanagisawa H (2021). Changes in health status, workload, and lifestyle after starting the COVID-19 pandemic: A web-based survey of Japanese men and women. Environ. Health Prev. Med..

[5] Yamada M, Kimura Y, Ishiyama D, Otobe Y, Suzuki M, Koyama S, Kikuchi T, Kusumi H, Arai H (2021). The influence of the COVID-19 pandemic on physical activity and new incidence of frailty among initially non-frail older adults in Japan: A follow-up online survey. J. Nutr. Health Aging.

[6] Steptoe A, Di Gessa G (2021). Mental health and social interactions of older people with physical disabilities in England during the COVID-19 pandemic: A longitudinal cohort study. Lancet Public Health.

[7] Piano C, Di Stasio E, Primiano G, Janiri D, Luigetti M, Frisullo G, Vollono C, Lucchini M, Brunetti V, Monforte M, Guglielmi V, Della Marca G, Evoli A, Marra C, Mirabella M, Quaranta D, Ricci E, Servidei S, Silvestri G, Bellavia S, Bortolani S, Bove F, Di Iorio R, Di Paolantonio A, Genovese D, Ialongo T, Lo Monaco MR, Marotta J, Patanella AK, Perna A, Petracca M, Presicce G, Riso V, Rollo E, Romano A, Romozzi M, Sancricca C, Scala I, Spagni G, Solito M, Tricoli L, Zinzi P, Calabresi P, Bentivoglio AR (2020). An Italian neurology outpatient clinic facing SARS-CoV-2 pandemic: Data from 2,167 patients. Front. Neurol..

[8] Schirinzi T, Landi D, Liguori C (2021). COVID-19: Dealing with a potential risk factor for chronic neurological disorders. J. Neurol..

[9] Manto M, Dupre N, Hadjivassiliou M, Louis ED, Mitoma H, Molinari M, Shaikh AG, Soong BW, Strupp M, Van Overwalle F, Schmahmann JD (2020). Management of patients with cerebellar ataxia during the COVID-19 pandemic: Current concerns and future implications. Cerebellum.

[10] Lebrasseur A, Fortin-Bedard N, Lettre J, Bussieres EL, Best K, Boucher N, Hotton M, Beaulieu-Bonneau S, Mercier C, Lamontagne ME, Routhier F (2021). Impact of COVID-19 on people with physical disabilities: A rapid review. Disabil. Health J..

[11] Fanciulli A, Wenning GK (2015). Multiple-system atrophy. New Engl. J. Med..

[12] Gilman S, Wenning GK, Low PA, Brooks DJ, Mathias CJ, Trojanowski JQ, Wood NW, Colosimo C, Durr A, Fowler CJ, Kaufmann H, Klockgether T, Lees A, Poewe W, Quinn N, Revesz T, Robertson D, Sandroni P, Seppi K, Vidailhet M (2008). Second consensus statement on the diagnosis of multiple system atrophy. Neurology.

[13] Yabe I, Soma H, Takei A, Fujiki N, Yanagihara T, Sasaki H (2006). MSA-C is the predominant clinical phenotype of MSA in Japan: Analysis of 142 patients with probable MSA. J. Neurol. Sci..

[14] Ozawa T, Revesz T, Paviour D, Lees AJ, Quinn N, Tada M, Kakita A, Onodera O, Wakabayashi K, Takahashi H, Nishizawa M, Holton JL (2012). Difference in MSA phenotype distribution between populations: Genetics or environment?. J. Parkinsons Dis..

[15] Ozawa T, Onodera O (2017). Multiple system atrophy: Clinicopathological characteristics in Japanese patients. Proc. Jpn. Acad Ser. B Phys. Biol. Sci..

[16] Horimoto Y, Aiba I, Yasuda T, Ohkawa Y, Katayama T, Yokokawa Y, Goto A, Ito Y (2002). Longitudinal MRI study of multiple system atrophy—When do the findings appear, and what is the course?. J. Neurol..

[17] Schrag A, Ben-Shlomo Y, Quinn NP (1999). Prevalence of progressive supranuclear palsy and multiple system atrophy: A cross-sectional study. Lancet.

[18] Camara A, Compta Y, Perez-Soriano A, Montagut N, Baixauli M, Maragall L, Ludena E, de Los L, Reyes JC, Peri-Cusi L, Fernandez N, Villote S, Ahuir M, Grau A, Caballol N, Buongiorno M, Pont-Sunyer C, Puente V, Giraldo DM, de Fabregues O, Garrido A, Navarro-Otano J, Painous C, Sanchez-Gomez A, Munoz E, Zaro I, Obiang D, Valldeoriola F, Lombrana M, Marti MJ (2021). Effects of COVID-19 pandemic and lockdown on people with multiple system atrophy participating in a therapeutic education program. Parkinsonism Relat. Disord..

[19] Gong Y, Chen Z, Liu M, Wan L, Wang C, Peng H, Shi Y, Peng Y, Xia K, Qiu R, Tang B, Jiang H (2021). Anxiety and depression in spinocerebellar ataxia patients during the COVID-19 pandemic in China: A cross-sectional study. J. Clin. Neurosci..

[20] Manto M, Dupre N, Hadjivassiliou M, Louis ED, Mitoma H, Molinari M, Shaikh AG, Soong BW, Strupp M, Van Overwalle F, Schmahmann JD (2020). Medical and paramedical care of patients with cerebellar ataxia during the COVID-19 outbreak: Seven practical recommendations of the COVID 19 cerebellum task force. Front. Neurol..

[21] Boggs D, Polack S, Kuper H, Foster A (2021). Shifting the focus to functioning: Essential for achieving sustainable development goal 3, inclusive universal health coverage and supporting COVID-19 survivors. Glob. Health Action.

[22] The World Health Organization (2001). International Classification of Functioning, Disability and Health: ICF.

[23] Haruyama K, Kawakami M, Miyai I, Fujiwara T (2022). The impact of the COVID-19 pandemic on three domains of functioning of ICF in participants with spinocerebellar degeneration and multiple system atrophy in Japan. Jpn. J. Rehabil. Med..

[24] Kroenke K, Spitzer RL, Williams JB (2003). The patient health questionnaire-2: Validity of a two-item depression screener. Med. Care.

[25] Kroenke K, Spitzer RL, Williams JB (2001). The PHQ-9: Validity of a brief depression severity measure. J. Gen. Intern. Med..

[26] Jacobi H, du Montcel ST, Bauer P, Giunti P, Cook A, Labrum R, Parkinson MH, Durr A, Brice A, Charles P, Marelli C, Mariotti C, Nanetti L, Sarro L, Rakowicz M, Sulek A, Sobanska A, Schmitz-Hubsch T, Schols L, Hengel H, Baliko L, Melegh B, Filla A, Antenora A, Infante J, Berciano J, van de Warrenburg BP, Timmann D, Szymanski S, Boesch S, Nachbauer W, Kang JS, Pandolfo M, Schulz JB, Melac AT, Diallo A, Klockgether T (2018). Long-term evolution of patient-reported outcome measures in spinocerebellar ataxias. J. Neurol..

[27] van Swieten JC, Koudstaal PJ, Visser MC, Schouten HJ, van Gijn J (1988). Interobserver agreement for the assessment of handicap in stroke patients. Stroke.

[28] Bouwstra H, Smit EB, Wattel EM, van der Wouden JC, Hertogh C, Terluin B, Terwee CB (2019). Measurement properties of the barthel index in geriatric rehabilitation. J. Am. Med. Dir. Assoc..

[29] Hachisuka K, Ogata H, Ohkuma H, Tanaka S, Dozono K (1997). Test-retest and inter-method reliability of the self-rating barthel index. Clin. Rehabil..

[30] Baker PS, Bodner EV, Allman RM (2003). Measuring life-space mobility in community-dwelling older adults. J. Am. Geriatr. Soc..

[31] Brooks R (1996). EuroQol: The current state of play. Health Policy.

[32] Tsuchiya A, Ikeda S, Ikegami N, Nishimura S, Sakai I, Fukuda T, Hamashima C, Hisashige A, Tamura M (2002). Estimating an EQ-5D population value set: The case of Japan. Health Econ..

[33] Watanabe H, Saito Y, Terao S, Ando T, Kachi T, Mukai E, Aiba I, Abe Y, Tamakoshi A, Doyu M, Hirayama M, Sobue G (2002). Progression and prognosis in multiple system atrophy: An analysis of 230 Japanese patients. Brain.

[34] Leonardi M, Meucci P, Ajovalasit D, Albanesi F, Cerniauskaite M, Invernizzi V, Lembo R, Quintas R, Sattin D, Carella F, Romito L, Soliveri P, Bussone G, D'Amico D, Maggi L, Mantegazza R, Raggi A (2009). ICF in neurology: Functioning and disability in patients with migraine, myasthenia gravis and Parkinson's disease. Disabil. Rehabil..

[35] Granlund M, Eriksson L, Ylven R (2004). Utility of international classification of functioning, disability and health's participation dimension in assigning ICF codes to items from extant rating instruments. J. Rehabil. Med..

[36] Raggi A, Leonardi M, Ajovalasit D, Carella F, Soliveri P, Albanese A, Romito L (2011). Disability and profiles of functioning of patients with Parkinson's disease described with ICF classification. Int. J. Rehabil. Res..

[37] Schirinzi T, Sancesario A, Castelli E, Bertini E, Vasco G (2021). Friedreich ataxia in COVID-19 time: Current impact and future possibilities. Cerebellum Ataxias.

[38] Colais P, Cascini S, Balducci M, Agabiti N, Davoli M, Fusco D, Calandrini E, Bargagli AM (2021). Impact of the COVID-19 pandemic on access to healthcare services amongst patients with multiple sclerosis in the Lazio region, Italy. Eur. J. Neurol..

[39] Manacorda T, Bandiera P, Terzuoli F, Ponzio M, Brichetto G, Zaratin P, Bezzini D, Battaglia MA (2021). Impact of the COVID-19 pandemic on persons with multiple sclerosis: Early findings from a survey on disruptions in care and self-reported outcomes. J. Health Serv. Res. Policy.

[40] van Uem JMT, Cerff B, Kampmeyer M, Prinzen J, Zuidema M, Hobert MA, Gräber S, Berg D, Maetzler W, Liepelt-Scarfone I (2018). The association between objectively measured physical activity, depression, cognition, and health-related quality of life in Parkinson's disease. Parkinsonism Relat. Disord..

[41] Wynia K, Middel B, van Dijk JP, De Keyser JH, Reijneveld SA (2008). The impact of disabilities on quality of life in people with multiple sclerosis. Mult. Scler..

[42] Gonzalez-Garces Y, Dominguez-Barrios Y, Zayas-Hernandez A, Sigler-Villanueva AA, Canales-Ochoa N, Hernandez Oliver MO, Ramírez-Bautista MB, Caballero-Laguna A, Arrufat-Pie E, Carrillo-Rodes FJ, Medrano-Montero J, Rodríguez-Álvarez Y (2021). Impacts of the COVID-19 pandemic on the mental health and motor deficits in Cuban patients with cerebellar ataxias. Cerebellum.

[43] Miyashita M, Narita Y, Sakamoto A, Kawada N, Akiyama M, Kayama M, Suzukamo Y, Fukuhara S (2009). Care burden and depression in caregivers caring for patients with intractable neurological diseases at home in Japan. J. Neurol. Sci..

[44] Matsumoto H, Kawagoe M, Hotta S (2021). Older adults used fewer home care services during the COVID-19 pandemic: Findings from a secondary analysis of an urgent survey In Japan. Ann. Geriatr. Med. Res..

[45] Terai H, Hori Y, Takahashi S, Tamai K, Iwamae M, Hoshino M, Ohyama S, Yabu A, Nakamura H (2021). Impact of the COVID-19 pandemic on the development of locomotive syndrome. J. Orthop. Surg. (Hong Kong).

[46] Han E, Tan MMJ, Turk E, Sridhar D, Leung GM, Shibuya K, Asgari N, Oh J, García-Basteiro AL, Hanefeld J, Cook AR, Hsu LY, Teo YY, Heymann D, Clark H, McKee M, Legido-Quigley H (2020). Lessons learnt from easing COVID-19 restrictions: An analysis of countries and regions in Asia Pacific and Europe. Lancet.

[47] Jain N, Hung IC, Kimura H, Lin GY, Jau W, Huynh KLA, Panag DS, Tiwari R, Prasad S, Manirambona E, Vasanthakumaran T, Amanda TW, Lin HW, Vig N, Thanh AN, Uwiringiyimana E, Popkova D, Lin TH, Nguyen MA, Jain S, Umar TP, Suleman MH, Efendi E, Kuo CY, Bansal SPS, Kauškale S, Peng HH, Bains M, Rozevska M, Tran TH, Tsai MS, Pahulpreet JS, Tai RZ, Khan ZA, Huy DT, Kositbovornchai S, Chiu CW, Nguyen THH, Chen HY, Khongyot T, Chen KY, Quyen DTK, Lam J, Dila KAS, Cu TN, My THT, Dung LA, Thi KON, Thi HAN, Thao MTD, Thi YC, Pham TT, Ariyoshi K, Smith C, Huy NT (2022). The global response: How cities and provinces around the globe tackled covid-19 outbreaks in 2021. Lancet Reg. Health Southeast Asia..

