# Supplementary material for: COVID-19 pandemic and the international classification of functioning in multiple system atrophy: a cross-sectional, nationwide survey in Japan
Source: Sci Rep. 2022 Aug 19;12:14163. doi: 10.1038/s41598-022-18533-w (PMC9389480; doi:10.1038/s41598-022-18533-w)
Supplement: Supplementary file 1 — Supplementary Information. [file 41598_2022_18533_MOESM1_ESM.docx]

COVID-19 pandemic and the International Classification of Functioning in multiple system atrophy: A cross-sectional, nationwide survey in Japan, J Neurol. Haruyama K, et al. Juntendo University, koshiroharuyama@gmail.com

**Multiple System Impairment Questionnaire (translated version)**

For each of the following symptoms, please select and circle one number from the four options (no impairment = 0, mild impairment = 1, moderate impairment = 2, severe impairment = 3).

| No. | Symptom items | Degree of impairment | | | |
| --- | --- | --- | --- | --- | --- |
|  |  | No | Mild | Moderate | Severe |
| 1 | **Ataxia / Incoordination**  Awkward limb movements  * Problems with timing, spacing, and grading of movements | 0 | 1 | 2 | 3 |
| 2 | **Muscle weakness / Muscle atrophy**  Loss of muscle strength (including disuse from inactivity) | 0 | 1 | 2 | 3 |
| 3 | **Muscle rigidity / Hypertonia**  State of stiffness or inflexibility  *muscle tone disorder with resistance to passive movement irrespective of posture and velocity | 0 | 1 | 2 | 3 |
| 4 | **Muscle spasticity**  Abnormal muscle tightness due to muscle contraction  *a velocity-dependent increase in muscle tone to passive movement | 0 | 1 | 2 | 3 |
| 5 | **Balance disorder**  Wobbliness or oscillation when standing or walking (including postural reflex disorder and/or frequent falls) | 0 | 1 | 2 | 3 |
| 6 | **Postural abnormality**  Excessively curved or tilted posture (including camptocormia or dropped neck) | 0 | 1 | 2 | 3 |
| 7 | **Decreased endurance**  Lack of physical fitness or stamina | 0 | 1 | 2 | 3 |
| 8 | **Fatigue**  Get tired easily (easily fatigued) | 0 | 1 | 2 | 3 |
| 9 | **Pain**  Physical pain sensation (for example, joint pain, low back pain, stiff shoulder) | 0 | 1 | 2 | 3 |
| 10 | **Numbness**  Showing or feeling numbness | 0 | 1 | 2 | 3 |
| 11 | **Sensory disturbance**  Dullness of the senses (for example, temperature, pain, touch, motor sensations, etc.) | 0 | 1 | 2 | 3 |
| 12 | **Tremor**  An involuntary quivering movement  *Different types of resting tremor, postural tremor, kinetic tremor, task-specific tremor, and intention tremor | 0 | 1 | 2 | 3 |
| 13 | **Involuntary movements**  Uncontrollable repetitive movement (not tremor)  *Different types of dyskinesia, myoclonus, tics, athetosis | 0 | 1 | 2 | 3 |
| 14 | **Orthostatic hypotension**  Including dizziness, syncope, faintness, and loss of consciousness (not due to other causes) | 0 | 1 | 2 | 3 |
| 15 | **Poor sleep**  Lack of sleep, insomnia, and other sleep deprivation | 0 | 1 | 2 | 3 |
| 16 | **Respiratory disturbance**  Feeling of dyspnea, difficulty breathing (including use of a respirator) | 0 | 1 | 2 | 3 |
| 17 | **Speech/dysarthria**  Hard to talk, articulatory disorder, dysphonia | 0 | 1 | 2 | 3 |
| 18 | **Dysphagia**  Hard to swallow or eat, including choking or aspiration | 0 | 1 | 2 | 3 |
| 19 | **Visual impairment**  Low visual acuity, ocular nystagmus, diplopia (double vision), and other visual problems | 0 | 1 | 2 | 3 |
| 20 | **Urinary dysfunction**  Frequent or difficult urination  *Pollakiuria, incontinence, urinary retention | 0 | 1 | 2 | 3 |
| 21 | **Voiding dysfunction**  Frequent or difficult bowel movements  *includes constipation and diarrhea | 0 | 1 | 2 | 3 |
| 22 | **Cognitive impairment / Dementia**  Decreased cognitive function (for example, memory and attention) and forgetfulness | 0 | 1 | 2 | 3 |

**Total Score /66**
